# Reproduction of group-housed sows

**DOI:** 10.1186/s40813-016-0033-2

**Published:** 2016-07-01

**Authors:** Olli Peltoniemi, Stefan Björkman, Dominiek Maes

**Affiliations:** 1 Department of Production Animal Medicine, Faculty of Veterinary Medicine, University of Helsinki, Helsinki, Finland; 2 Unit of Porcine Health Management, Department of Reproduction, Obstetrics and Herd Health, Faculty of Veterinary Medicine, Ghent University Belgium, Ghent, Belgium

**Keywords:** Sow, Reproduction, LH, Group housing, Grouping, Stress, Feeding

## Abstract

The sow is a social animal in her behavior throughout the reproductive cycle. An exception to her preference for being a part of a social group occurs one to two1–2 d days prior to farrowing, when she separates from her group and seeks for isolation in order to build up a nest. She then spends the first week or two with her piglets, mainly in the nest. After this short period of separation of 1–2 weeks, she brings her litter with her and rejoins the group. In modern intensive pig production, the sow is often restricted to an individual cage for lactation and, in many European countries, she may still spend additional periods in stalls during pregnancy. In the intensive production, isolation of the sow from the rest of the group is therefore a relatively long period of six to ten6-10 weeks, which creates a challenge for the social memory of the sow. While grouping of sows during lactation is an interesting option, until now this is encountered mostly in organic or otherwise extensive farming systems, such as outdoor farming. However, the present society is asking for more animal friendly models of production and there appears to be more need for studies of group housing issues during lactation. Grouping of sows after weaning causes stress, which imposes risks for fertility. Thus, timing of grouping is probably very critical. It is well documented that the embryonic period of the pregnancy, lasting up to Day 35, is more vulnerable for loss of pregnancy than the subsequent fetal period. There are indications that stress of grouping may cause some harm to vitality parameters of blastocysts already while at the site of fertilization in the oviduct. Later on, during the critical periods of maternal recognition of pregnancy, endocrinological models testing maintenance of pregnancy suggest that chronic stress lasting for more than two2 days may cause abortion and loss of the whole litter. However, the sow may be resistant, in terms of her reproductive function, to acute stress lasting for hours or up to a day. In conclusion, grouping of sows during lactation may be of interest in the future. At present, issues of group housed sows after weaning and early pregnancy seem to be of most practical relevance. Chronic stress of sows lasting for more than two2 days may lead to loss of the whole litter.

## Background

**Fig. 1:**
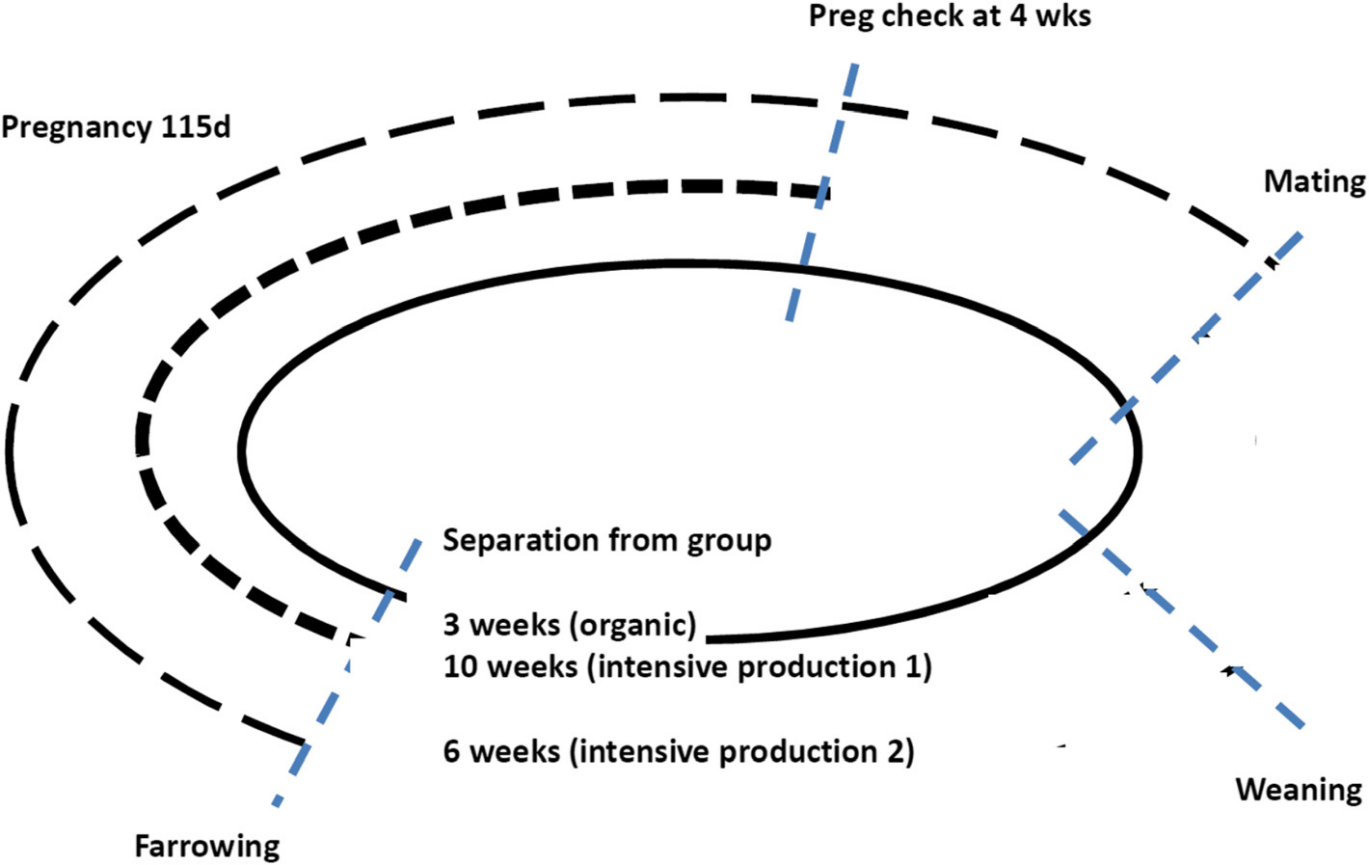
Illustration of social separation and thereby isolation of the sow from her group in different production scenarios; in organic farming (solid line), the separation lasts for three weeks only, whereas in the most common two intensive production scenarios (two sets of dashed lines), the separation lasts alternatively for 6 weeks or 10 weeks. In organic farming sows are grouped in week 2 of lactation, whereas in intensive production 1, sows are in crates for 4 weeks after AI. In intensive production 2, sows are grouped upon mating

In nature, sows spend almost their whole life in groups; -the only exception is an isolation of about nine to eleven9-11 days around the time of farrowing (Fig. 1; [9, 10]). This is fairly close to the three3 week isolation practiced in the organic farming. On the contrary, in modern intensive housing practices, isolation for six6 toor ten10 weeks appears more common. This period spans the limits of the social memory of a sow. In addition, reunion of sows into a group during lactation may provide with a better chance to induce an early oestrus and insemination with reasonable outcome [22]. This would provide the farmer with a chance to speed up the production cycle;-and a modest reduction in litter size. The reduction in litter size may be considered as a favorable outcome in extensive environment, where the responsibility of taking care of the litter lies more on the sow than on human handling. This paper will focus on the effect of isolation and group housing on reproductive physiology of the sow. The effects of group housing on sow health is discussed in an accompanying paper (Maes et al. 2015, unpublished).

## Group housing during lactation

There appears to be a growing need for animal friendly models of grouping sows in Europe [11, 18, 22]. One phase of production where keeping sows in groupssuch a development appears possible is keeping sows in groups duringthe lactation [5]. Continuous exposure of the sow to her piglets may be stressful for the sow in a traditional lactation setup with individual housing. In group lactation systems [8], sows can get away from the piglets and the burden of lactation may be somewhat alleviated. Reproduction performance might, however, be influenced by group-housing. In a Swedish study, where sows were housed in groups of four between the third and seventh week for weeks 3 to7 of lactation, approximately every second sow came into oestrus during lactation [8]. Of those that came into oestrus, only 50 % were showing signs of standing reflex. Moreover, lactational oestrus seems to be more common in older sows, which may be connected to more intensive mothering behavior observed in younger sows. While in groups during lactation, sows may not be subject to an efficient boar contact and heat detection may not be optimal. Further information is needed to help farmers to set up a production environment where group housing of lactating sows may become a practical option in the future. In principle, grouping sows by having sows in groups during lactation may avoid, stress related to grouping sows laterat whichever point after weaning. may be avoided. However, lactation groups may be too small for forming reasonable groups after weaning for the pregnancy unit.

## Grouping sows after weaning and AI

In order to avoid mixing of sows after insemination, one practical option is to form a group right after weaning. This practice has clear advantages:; 1) establishing a rank in the group is supposed to take about 24 h;-if the rank is established already before fertilization, the risk related to aggression during the embryonic period is absent;, 2) social contacts between females are known to advance onset of oestrus;, and 3) it has been stated that social interaction between females are considered as “positive stress”, which is supposed to stimulate oestrus induction of oestrus [5]. However, there is more labor related to moving of sows to the boar or vice versa for oestrus detection and artificial insemination (AI). In addition, there is some evidence to suggest that social interactions may suppress oestrus signs in subordinate sows [27]. Proper grouping of sows according to size and age may overcome this problem. Because social interactions may suppress oestrus signs in subordinate sows [27] and because of duration of the standing reflex of maximal 10 minutes [13], it is advisable to separate individual sows or a small group of sows from the rest of the group for heat detection and artificial insemination. This can be done, i.e. by taking the sow/small group of sows out of the pen and moving them to the boar. In any case, the separation of sows requires more labor.

Group housed sows at AI can show oestrus freely compared to sows restricted to stalls [6] which may improve the possibilities for observing oestrus behavior and reduce the risk of inseminating a sow that is not in standing oestrus [5]. Another benefit is that sows may easily be exposed to several boars instead of one only which may potentiate the effect of boar stimulation, triggering the standing reflex better. This practical assumption stems on the fact that if grouped already at weaning, it is sows that move to the boars in small groups to the front of the boars. It should also be noted that social interaction during establishment of a dominance order of the group and/or exhibition of oestrus behavior are temporal behaviors of sows that may require more space per sow as compared to behavior expressed by pregnant sows. Furthermore, the social interaction may be beneficial for expression of stronger signs of oestrus, alleviating management of oestrus detection [5]. Sows that are at the borderline of having a standing reflex can be detected more easily in a group, preferably in presence of a boar.

Benefits of having sows in a group at the time of oestrus and AI are: 1) that sows can interact and show heat freely, which improves the expression of oestrus behavior and alleviates the detection of sows that are in heat; 2) that interaction between sows can boost the expression of oestrus behavior in sows that are at the borderline of having a standing reflex; 3) that the risk of inseminating a sow that is not in standing heat is reduced. On the other hand, subordinate sows might express oestrus behavior not as much in a group as in individual housing. Therefore, it is advisable to separate individual sows or a small group of sows from the rest of the group for heat detection and artificial insemination. This can be done, i.e., by taking the sow/small group of sows out of the pen and moving them to the boar. In conclusion, the advantages of having sows in a group at oestrus clearly outweigh the disadvantages, as long as sows are grouped early enough, i.e., immediately after weaning. Still, it requires more labor because of separation and moving of sows for AI. Furthermore, it should be noted that social interaction during establishment of a dominance order of the group and/or exhibition of oestrus behavior are temporal behaviors of sows that may require more space per sow as compared to behavior expressed by pregnant sows stable group situation during pregnancy.

Regarding reproductive performance of the sow, an increased risk of repeat breeding has been associated with group housing after weaning in some studies earlier [17, 20]. However, in a large scale comparison, having all sows grouped after weaning did not appear to have a detrimental effect on reproductive performance [5]. Furthermore, when different housing systems were reviewed [11], increased rate of returns to oestrus was associated by level of management and expertise rather than group housing per se [11]. In conclusion, sows may exhibit oestrous behavior better when in groups already after weaning. However, in order to avoid potential problems with maintenance of early pregnancy and maintain a high level of reproductive performance, good management skills with sow groups are needed. In addition, AI work of sows in groups may require additional resources, which may imply that management becomes more costly to the industry.

## Group housing of pregnant sows

Group housing of pregnant sows has been compulsory since year 2013 in Europe and it has been widely adopted in other parts of the world such as the US, Canada, New Zealand and Australia [23]. However, transition to group housing has been somewhat slow with farmers being either reluctant to invest in new systems requiring more space or lacking confidence towards group housing. Replacing stalls with less space consuming structures may reduce need to build up completely new facilities for group housing. In management of group housing, producers are facing new challenges. Inter-sow confrontations mostly relate to feeding time (also after mixing). The confrontations may present a risk factor to fertility with an apparent tendency towards more early loss of embryos and increased rate of repeat breeding after a prolonged oestrus to oestrus interval [17]. Housing and management needs to be considered carefully to reduce this risk. The housing system should be designed in a way that allows sows to show proper social behaviour, and that gives subordinate sows a possibility to escape from confrontation with another sow. It is also important to make sure that all sows can eat undisturbed. Regarding space allowance, a number of studies suggest that as a general rule sows should be provided with 2.5–3.5 m^2^ per sow in order to reach a reasonably high farrowing rate [11]. In addition, feeding enough bulk and fibre and provide the pen with areas to escape helps to alleviate aggressive behaviour problems of housing [21]. Furthermore, it is regularly reported that having a teaser boar in the pregnant sow group calms the group down and reduces aggression among sows. For the group size, small groups may be more natural for sows. However, large dynamic groups of up to 300 sows may provide the farmer with satisfactory fertility results given that there is enough space in the group and if they are otherwise well managed. There is some evidence to suggest that during 2–3 weeks after fertilization, when implantation and establishment of pregnancy occurs, is the most vulnerable period subject [25]. However, to the knowledge of the authors, the first 3 weeks of pregnancy, based on the studies discussed below, need all to be included as a vulnerable period where loss of embryos and pregnancies may occur more easily than at other times. In conclusion, successful management of sow groups during early pregnancy requires adequate space and management, including bulk feeding to support the eating behaviour, satiety and comfort of the sow.

## Stress and maintenance of pregnancy

**Fig. 2:**
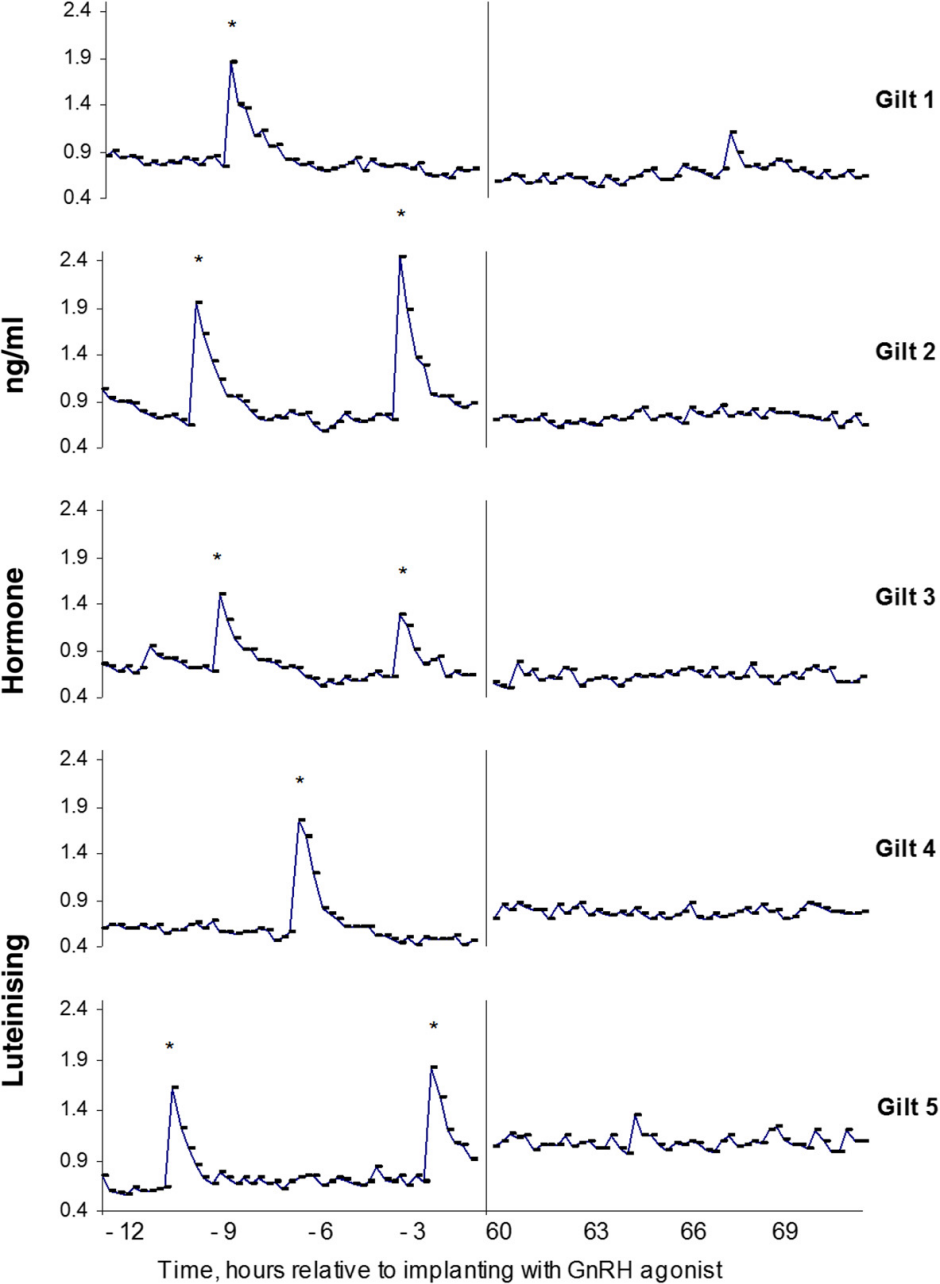
Using a GnRH agonist model to study the effect of LH pulsatility on maintenance of pregnancy in gilts. In all gilts inseminated between days 0–21, pregnancy was interrupted, whereas after d 21, pregnancy was interrupted in a proportion of gilts only. The model shows importance of LH pulsatility for maintenance of pregnancy during the embryonic period. Data from [19]

We have tried to evaluate the effects of stress on CL function and maintenance of pregnancy in group housed gilts and sows in a series of studies. In this line of research, stress effects are thought to be mediated via the hypothalamo-pituitary axis. It has been well documented that LH secretion is subject to stress effects, mainly through cortisol mediated stress responses [14]. LH is likely to carry over the stress effects on to the CL function, which in turn is a basis for the endocrine milieu within the uterine lumen for successful establishment and maintenance of pregnancy. In pigs, development of the CL after ovulation and the secretion of progesterone occur independently of LH input from the pituitary, at least until 10–12 days after ovulation [19]. Hypophysectomy on the day after oestrus or mating does not prevent the development of normal-sized, progesterone-secreting CL by day 12 after oestrus [1], but CL do regress between days 16 and 20 in pregnant, hypophysectomised sows. Meduri et al. [16] showed that at 48 h after follicle rupture, there is a marked decrease in the density of LH receptors in luteal cells, and 6 days after ovulation the receptor density seems to increase again. These findings indicate an LH-independent and LH-insensitive window during early development of the corpus luteum. More recent studies by our group have approached the role of LH on the maintenance of luteal tissue using three different models (Fig. 2). First, pregnant gilts received GnRH agonist implants to down regulate GnRH receptors and suppress LH pulsatility [19]. Second, active and passive immunisation against GnRH was used to reduce LH pulsatility in the early pregnant gilt [24]. Third, a GnRH antagonist was used to directly down regulate LH pulses [30]. Based on these models, beyond days 10 and 12 of pregnancy, support of the CL by LH does become important, although in some studies it seems that reduction in gonadotrophic support has to be severe and chronic to result in luteal regression and pregnancy failure. LH secretion during the luteal phase of the oestrous cycle and during early pregnancy is characterized by a lower frequency of greater amplitude LH pulses. Chronic treatment with a GnRH agonist from days 14 to 21 of pregnancy abolished LH secretion and resulted in a decrease in progesterone secretion and loss of pregnancy in all sows at around 15 days after the start of treatment [19]. Similarly, Easton et al. [3] observed a decline in progesterone between 13 and 21 days after implantation with a slow-release agonist of GnRH (at oestrus), which is 3 to 11 d after CL have started to become sensitive to LH. The use of a GnRH agonist by its nature initially increases LH release before down-regulating LH pulses, and may also cause some extra luteal tissue to be formed, delaying the suppression of LH and effects on luteal function. In contrast, single injection with a GnRH antagonist between days 14 and 19 after ovulation resulted in a more immediate disruption of LH secretion for a period of 2.7 days, on average, and loss of pregnancy in three of 15 sows [29]. Active or passive immunisation against GnRH [24] had a more immediate effect with a reduction in progesterone within 2 to 4 days, and luteal failure evident within 7–10 days from immunization. In the immunisation model, none of the sows maintained pregnancy. Interestingly, immunization at day 10 of pregnancy seemed to cause a reduction in progesterone and failure to establish pregnancy before total luteal failure occurred, whereas immunization at day 20 of pregnancy resulted in total luteal failure before abortion occurred. The models described above indicate that a strong suppression of LH that lasts 3 to 5 days will result in luteal failure and as a consequence result in no pregnancy being established or abortion, depending on the stage of pregnancy. As a conclusion, there is enough evidence to state that LH secretion or a minimum of LH pulses is important to support CL and maintenance of early pregnancy in the pig, beyond day 12 after fertilization. In conclusion, under group housing, if a stress factor is suppressing LH secretion and thereby CL function for longer than 2 days, regression of CL may occur causing disruption of pregnancy and loss of the whole litter.

## Feeding issues of pregnant group housed sows

Sows may show aggressive behaviour either at the establishment of rank or when fighting for resources such as feed. Under normal production circumstances, feed restriction of up to 50 % is applied to group housed pregnant sows. Earlier, it has been indicated that feed restriction of pregnant sows can be considered as a cause for stereotypic behaviour in the pig [26]. A competitive feeding system, such as floor or trough feeding may present sows with a chronic stress situation, where they are subjected to stress for more than the threshold time of 2 days discussed above. If ad libitum feed is given, sows may have up to 13 occasions of feeding within a 24 h period, which may approach the feeding behaviour seen in the nature. Ad libitum feeding therefore seems as an animal friendly way of feeding, reducing stereotypic behaviour related to feeding times [23]. Common systems, such as free access stalls and electronic sow feeders, have been developed to control excessive competition for food-these systems may work satisfactorily if feeding and water supply management is otherwise well managed. However, sows prefer to synchronise their feeding, and individual feeders always introduce more competition, and thus a risk for increased aggression. It is a generally accepted as principle in the pig industry, that sows should remain in as stable as possible condition throughout the production cycle [4]. This principle stems from the idea that sows should lose weight as little as possible during lactation and, on the other hand, gain weight only moderately during pregnancy. With the increase in litter size and in the number of fetuses, pushing the uterine capacity of contemporary sows to the limit, the challenge of not losing too much weight during lactation is becoming increasingly difficult [7]. The energy equation appears especially challenging for the low parity sows, as they are supposed to gain weight and grow during the first two parities. It has been shown that abundant feeding of low parity group housed sows may reduce the risk of interruption of pregnancy [12, 30, 31]. Motivation for food is highest during the first 7 weeks of pregnancy as shown by an ad libitum feed intake profile along the duration of pregnancy [28]. Furthermore, studies on seasonal infertility have indicated that an abundant feeding during early pregnancy may alleviate seasonal effects on pregnancy rates in the sow [14, 15, 30]. It has been reported that adding large quantities of fibre in the diet of the pregnant sow decrease stereotypies and prolong resting time [2]. In conclusion, in group housed sows, a good way of reducing stereotypies is to increase the fibre content and overall volume of feed and decrease the energy content. This seems like a solution to many practical problems related to group management of pregnant sows.

## Conclusions

Grouping issues during lactation maybe of increasing interest in the future. At present, improving systems for group housed sows after weaning and early pregnancy seem to be of most practical relevance. Endocrinological models to manipulate LH secretion suggest that chronic stress of sows lasting for more than 2 days during the embryonic phase of pregnancy may lead to cessation of luteal function and loss of the whole litter. Management issues such as correct grouping of sows according to age and size right after weaning, adequate space allowance of more than 2,5 m^2^ per sow, use of teaser boar in pregnant sow groups, using feed stuff of higher volume with more fibre and avoiding competition at feeding and avoiding stressors such as mixing sows during the first 3 weeks of pregnancy may all improve fertility of group housed sows.
